# Structure Insights Into Photosystem I Octamer From Cyanobacteria

**DOI:** 10.3389/fmicb.2022.876122

**Published:** 2022-05-06

**Authors:** Ming Chen, Yujie He, Dongyang Liu, Lijin Tian, Pengqi Xu, Xuan Liu, Yihang Pan, Shuqi Dong, Jun He, Ying Zhang

**Affiliations:** ^1^The Seventh Affiliated Hospital, Sun Yat-sen University, Shenzhen, China; ^2^Center for Cell Fate and Lineage (CCLA), Bioland Laboratory (Guangzhou Regenerative Medicine and Health Guangdong Laboratory), Guangzhou, China; ^3^Photosynthesis Research Centre, Institute of Botany, Chinese Academy of Sciences (CAS), Beijing, China; ^4^Center for Cell Lineage and Development, Guangzhou Institutes of Biomedicine and Health, Chinese Academy of Sciences, Guangzhou, China

**Keywords:** cyanobacteria, photosystem I, hexamer, octamer, single particle

## Abstract

The diversity of photosystem oligomers is essential to understanding how photosynthetic organisms adapt to light conditions. Due to its structural and physiological significance, the assembly of the PSI supercomplex has been of great interest recently in terms of both chloroplast and cyanobacteria. In this study, two novel photosystem I supercomplexes were isolated for the first time from the low light incubated culture of filamentous cyanobacterium *Anabaena* sp. PCC 7120. These complexes were defined as PSI hexamers and octamers through biochemical and biophysical characterization. Their 77K emission spectra indicated that the red forms of chlorophylls seemed not to be affected during oligomerization. By cryo-EM single-particle analysis, a near-atomic (7.0 Å) resolution structure of a PSI octamer was resolved, and the molecular assemblies of a stable PSI octamer were revealed.

## Introduction

Photosystem I (PSI) is a light-driven plastocyanin: ferredoxin oxidoreductase with a quantum efficiency close to 100%—probably the most efficient photoelectron converter in nature (Amunts and Nelson, 2009). The function of this bio-macromolecule is highly conserved in cyanobacteria, algae, and plants, yet it carries complicated structural diversities, especially in cyanobacteria. It has been acknowledged for years that the chloroplast PSI-LHCI complex can only form a monomeric state. And this classic theory was challenged by an observation of the spinach thylakoid membrane with atomic force microscopy (AFM) in which PSI-LHCI complexes tend to assemble around each other to form a dimeric supercomplex in dark conditions (Wood et al., 2018). Additionally, an atomic-resolution (2.97 Å) structure of the novel dimeric PSI-LHCI supercomplex was recently resolved in low light-adapted green alga *Chlamydonomas reinhardtii* (Naschberger et al., 2021). Things are more complicated in cyanobacteria in which the PSI complex is assembled as a trimer in most species (Boekema et al., 1987; Kruip et al., 1994; Jordan et al., 2001); meanwhile, the dimer and tetramer have been identified in some heterocyst-forming cyanobacteria and their close unicellular relatives (Li et al., 2014, 2019; Watanabe et al., 2014; Zheng et al., 2019; Chen et al., 2020). Recently, high-resolution structures of PSI monomers with full physiological function were also resolved in some species of unicellular cyanobacteria (Netzer-El et al., 2018; Çoruh et al., 2021). All these studies have indicated that the dynamic oligomeric states of the PSI complex are essential to helping photosynthetic organisms adapt to changing environments, especially under stress conditions, such as limited light energy supply.

Moreover, by using blue-native PAGE, Li and co-workers observed a possible PSI hexamer in *Chroococcidiopsis* sp. PCC 6712 upon thylakoid membrane extraction (Li et al., 2019). Before that, a projection of two associated PSI tetramers was spotted with single particle analysis in filamentous cyanobacterium *Anabaena* sp. PCC 7120 (here after *Anabaena* 7120; Watanabe et al., 2014). Due to the key role of PSI oligomer states in understanding the photosynthesis mechanism, molecular evidence and structure insights into those higher-ordered PSI oligomer complexes are of significant interest and are highly desired now. Here, two novel oligomer states of the PSI complex were isolated from the low light incubated culture of *Anabaena* 7120. The pure molecules were set into biophysical analysis, and the existence of higher-ordered PSI supercomplexes in cyanobacteria was ultimately demonstrated. By employing cryo-EM single-particle analysis, one of these PSI supercomplexes was subjected to high resolutions structure determination in which a novel molecular interface for PSI complex assembly was detected.

## Materials and Methods

### Species and Growth Conditions

Filamentous cyanobacteria *Anabaena* 7120 (FACHB-418), were purchased from the Institute of Hydrobiology, Chinese Academy of Sciences. Cyanobacterial cells were cultured at 20°C–30°C in BG-II (+N) media. The liquid culture in the flask was incubated under illumination with fluorescent lamps (5–50 μE m^−2^ s^−1^).

### Membrane Protein Complexes Isolation

For membrane complex extraction, 150 ml liquid cultures at the logarithmic phase (A_730_ = 1.0–1.3) were harvested at 4,000 *g* centrifugation under 4°C, and cells were washed once in 15 ml pre-cooled buffer A (50 mM HEPES, 10 mM MgCl_2_, 5 mM CaCl_2_ and 15 mM NaCl). Cells were then resuspended in 10 ml buffer A and treated with a cycle of 15 min frozen in −80°C refrigerator and 10 min melting at room temperature. Treated cells were harvested at 4,000 *g* centrifugation under 4°C and resuspended in 1.5–3 ml buffer A, and 10% (w/v) of β-DDM were supplemented to the given final concentration for membrane solubilization and complex extraction. The mixture was incubated on ice for 30 min, and insolubilized materials were removed by 14,000 *g* centrifugation under 4°C for 15 min. The supernatants were harvested for supercomplex isolation. For fluorescence emission spectra samples, solubilized supernatant was subjected to sucrose gradient ultracentrifugation (10%–50% (w/v) sucrose in buffer A with 0.03% (w/v) digitonin) at 35,000 rpm (sw41 Ti rotor, Beckman Coulter) for 16 h at 4°C. The band containing PSI tetramers and lower bands were collected with a syringe and directly used for spectroscopic analysis. For single-particle samples, supercomplexes in solubilized supernatants were isolated and collected through a Blue-Native PAGE and re-extraction method.

### Blue-Native PAGE and Re-extraction

BN-PAGE was performed in a cold room or a large ice bucket to maintain the low temperature (4°C) during electrophoresis. A Bio-Rad Mini-PROTEAN Tetra Cell (1658006) system was used for mini gel casting and electrophoresis running. The reagents for gel casting, the anode, and cathode native PAGE buffers were prepared according to Wittig’s protocol (Wittig et al., 2006). A gradient generator was used to prepare the acrylamide gradient gel. In this study, 3%–10% (w/v) acrylamide gel was selected according to the large molecular weight of megacomplexes. In most cases, 4%–13% (w/v) gels are feasible to separate proteins with molecular weights ranging from 10 to 3,000 kDa, and 3%–13% (w/v) gels are appropriate for separating 10–10,000 kDa protein molecules. Electrophoresis power was generated using a Bio-rad PowerPac TM Basic Power Supply (1645050). In the first stage, cathode buffer B was used, and the power supply was set to 135 V for electrophoresis. Stage two involved exchanging cathode buffer B with cathode buffer B/10 when the samples were moved into a separate gel (approximately 30 min). The running was maintained at 135 V for another 1.5–2 h until acceptable separation efficiency was obtained.

For the re-extraction of proteins, target bands were excised immediately after electrophoresis and stored in an ice-cooled Eppendorf tube. Gel bands were manually homogenized with a glass rod directly into the tube to minimize the loss of protein samples. To prevent heat damage to the protein complex, 10 cycles of 30 s on and 60 s off were programmed for homogenization. The gel slurry was then mixed with re-extraction buffer (buffer A with 0.03% digitonin), and the gel debris was removed by centrifugation at 14,000 × *g* for 15 min at 4°C. The supernatant was subjected to a second run of BN-PAGE for purity and integrity check and concentrated to qualified concentration with a 100 kDa cut-off Millipore membrane filter (UFC5100BK) for single-particle analysis.

### Cryo-EM and Image Processing

Three microliters of purified PSI supercomplex were applied onto glow-discharged holey carbon grids (Cu Quantifoil R1.2/1.3) at a protein concentration of approximately 2.5 mg/ml, prior to vitrification using a Vitrobot MKIV (3.0 s blot 5, 4°C, 100% humidity). The images were collected in 17 different sessions on a 200 kV FEI Titan Krios electron microscope (50 μm C2 aperture). A Gatan K3-Summit detector was used in counting mode at a magnification of 45,000 (yielding a pixel size of 0.88 Å), and a dose rate of 30 electrons per pixel per second. Exposures of 1.618 s (yielding a total dose of 63 eÅ^−2^). SerialEM was used to collect a total of 23,491 images, which were fractionated into 27 movie frames with defocus values ranging from 1.5 to 2.5 μm. All datasets were processed separately by using the same procedure. Collected micrographs were corrected for local-frame movement and dose-filtered using Motioncor2 (Zheng et al., 2017). The contrast transfer function parameters were estimated using Gctf (Zhang, 2016). In total, 184,882 particles were auto picked using warp, and they were subjected to reference-free two-dimensional class averaging in Relion 3.0.8 (Zivanov et al., 2018). After 2D classification, 100 k good particles were generated in which top-views were divided into 10 subsets. Each subset was combined with the side-view separately for the 3D map refinement in cryoSPARC (Punjani et al., 2017), yielding a map at an overall resolution of 7.0 Å. No symmetry was applied during the final processing, as it did not improve the map. The reported resolutions are based on 3D refinement by applying a 0.143 criterion on the FSC between reconstructed half-maps. All figures were generated using PyMOL (DeLano, 2002) and Chimaera X (Goddard et al., 2018). A local resolution map was generated using the cryoSPARC.

### Negative Staining EM and Data Processing

The purified PSI tetramer and supercomplexes were adjusted to 0.15, 0.19, and 0.27 mg/ml protein, respectively. In all cases, 3 μl of the protein solution was loaded on the glow-discharged copper grid. The excess protein solution was blotted with filter paper. The grid was washed twice with distilled water and then stained with 0.75% (w/v) uranyl formate for 30 s. The stain solution was blotted and the grid was dried in air. The grids were examined using a Tecnai G2 Spirit electron microscopy under 120 kV accelerating voltage. Micrographs were recorded with a CCD camera at a normal magnification of 49,000× with a variation of defocus values from −1.5 to −2.5 μm, resulting in a pixel size of 4.3 Å at specimen level. In total, 34, 16, and 18 images were manually collected for the PSI tetramer, supercomplex 1 and supercomplex 2, respectively. In total, 359, 2,518, and 822 particles were manually picked and were proceeded to a reference-free two-dimensional class averaging using Relion 3.0.8.

### Steady-State Fluorescence Spectroscopy

Fluorescence emission spectra were recorded on a FLS1000 Photoluminescence Spectrometer (Edinburgh). The sample OD at the maximum emission in the red region of the spectrum was adjusted 0.05 for fluorescence measurements. A bandwidth of 3 nm was used for both excitation and emission, and the step size was 1 nm. An optical long-pass filter (590 nm) was placed before the detector to remove scattered light.

## Results and Discussion

### Discovery of Higher-Ordered PSI Complexes

Blue-native PAGE has been recognized as a powerful tool to investigate thylakoid membrane complexes (Wittig et al., 2006; Järvi et al., 2011). The bands corresponding to complexes of high molecular weight suggest the existence of higher-order oligomeric states of photosynthetic membrane complexes currently undefined, especially for PSI assembled hetero- or homo- complexes (Zhang et al., 2010; Järvi et al., 2011; Kouřil et al., 2018).

**Figure 1:**
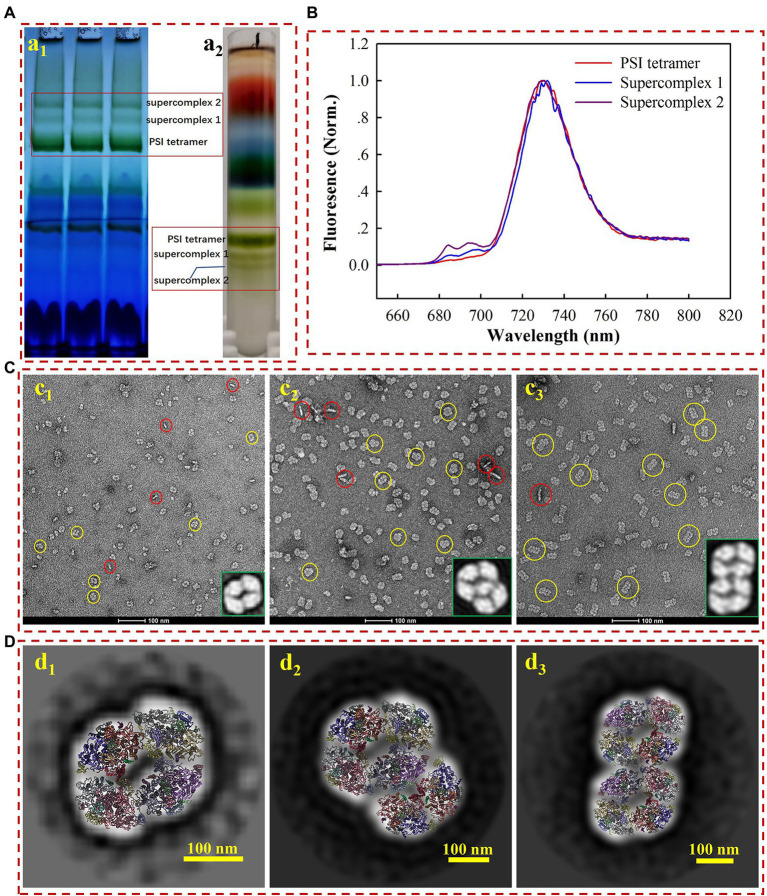
Biochemical **(A)** and biophysical **(B–D)** characterization of the PSI tetramer, supercomplex 1, and supercomplex 2. **(a**_**1**_**)** Membrane crude extracts were loaded on BN-PAGE at a total amount of 9–12 μg chlorophyll a for each lane; **(a**_**2**_**)** membrane crude extracts were loaded on sucrose gradient ultracentrifugation for the separation of PSI tetramer and supercomplexes. **(B)** Low temperature (77 k) fluorescence emission detection of PSI tetramer (red line), supercomplex 1 (blue line), and supercomplex 2 (purple line). **(C)** Negative staining electron microscopy image of PSI tetramer **(c**_**1**_**)** supercomplex 1 **(c**_**2**_**)** and supercomplex 2 **(c**_**3**_**)**. The yellow and red circles indicate the top and side views, respectively. The enlarged image in the right corner shows the 2D averages of the most contributed particles. **(D)** The average 2D model-fitted maps of the PSI tetramer **(d**_**1**_**)** supercomplex 1 **(d**_**2**_**)** and supercomplex 2 **(d**_**3**_**)**.

As shown in Figure 1Aa_1_, two undescribed complexes (supercomplex 1 and 2), both larger than the PSI-tetramer (1,400 kDa), were extracted from *Anabaena* 7120 using 1% (w/v) β-DDM and subsequently separated on BN-PAGE gel. The naturally green color of the bands indicates that these are chlorophyll-containing complexes, most likely the PSI-assembled complexes. To verify this hypothesis, the complexes were separated and harvested through sucrose gradient ultracentrifugation. The fraction scattering in sucrose gradients (Figure 1Aa_2_) is highly consistent with the gradient gel results in the BN-PAGE experiment (Figure 1Aa_1_). The fractions corresponding to supercomplex 1 and supercomplex 2 were collected and subjected to low temperature (77 K) fluorescence spectra measurement under the excitation of the 430 nm, and the PSI tetramer fraction was also harvested for comparison. As shown in Figure 1B, the emission spectra of supercomplexes 1 and 2 peaked at ~730 nm, largely overlapping with the one of PSI tetramer. Their emission spectra showed that the lowest energy level of quantum yield of chlorophylls in PSI remained ~730 nm, indicating that the red forms of chlorophylls were not energetically altered. This fluorescence spectral characteristic strongly suggests that these two complexes are both PSI homogenous supercomplexes (Watanabe et al., 2014; Lamb et al., 2018; Chen et al., 2020).

For a closer look at the molecules, the complexes were subjected to negative staining electron microscopy (EM) analysis. While the homogeneity of particles from all three samples is acknowledged (Figures 1Cc_1_–c_3_), the remarkable diversity in particle size from different samples is also revealed by negative staining EM. We subsequently used the single particle analysis (SPA) approach to check the oligomeric states of various samples. Using the high-resolution structure of the PSI tetramer as a reference (Kato et al., 2019; Zheng et al., 2019; Chen et al., 2020), particles in micrograph Figure 1Cc_1_ are generated as tilted views of the PSI tetramer. Over 90% of the particles presented a top-view (yellow circles) orientation parallel to the thylakoid membrane plane, while about 9.5% of them are side views (red circles). The contribution of the top and side views is consistent with those previously reported (Watanabe et al., 2014). For the samples of supercomplex 1 and supercomplex 2, the particle morphology in the images indicated that these proteins are PSI higher-ordered oligomeric complexes (Figures 1Cc_2_,c_3_).

To further characterize the molecular architectures, three datasets were collected for the PSI tetramer, supercomplex 1, and supercomplex 2, respectively, from which particles were manually picked. Particles were then used for pre-processing and 2D classification, and the most dominant averages are shown as the enlarged part in the bottom right corner of Figures 1Cc_1_–c_3_. From these closer views, it is strongly suggested that supercomplex 1 is a PSI hexamer consisting of a tetramer and a dimer, while supercomplex 2 is a PSI octamer formed by two assembled tetramers, which agrees with the previous results (Watanabe et al., 2014). This conclusion is supported by a rigid fitting of PSI tetramer and dimer models over the 2D map of the complexes, respectively (Figure 1D). By comparison to the PSI tetramer, the PSI hexamer can be identified as a complex arranged with a tetramer and dimer with substantial confidence. However, it is ambiguous to identify the interface for the linkage of the PSI tetramer and dimer in such a low-resolution map. Furthermore, from the fitted model (Figure 1Dd_2_), the PsaL-PsaA, PsaK-PsaK, and PsaM-PsaF interfaces should be addressed in further studies providing high-resolution details. The PSI octamer revealed in Figure 1Dd_3_ is a supercomplex assembled though a ‘side by side’ arrangement of two tetramers, the fitted model reveals the possible interfaces of PsaA-PsaF, PsaK-PsaK, and PsaB-PsaK. Studies providing a high-resolution structure would also be appreciated to investigate the detailed molecule basis for the intra- and inter-connection of these supercomplexes.

### Cryo-EM Structure of the PSI Octamer Complex

**Figure 2:**
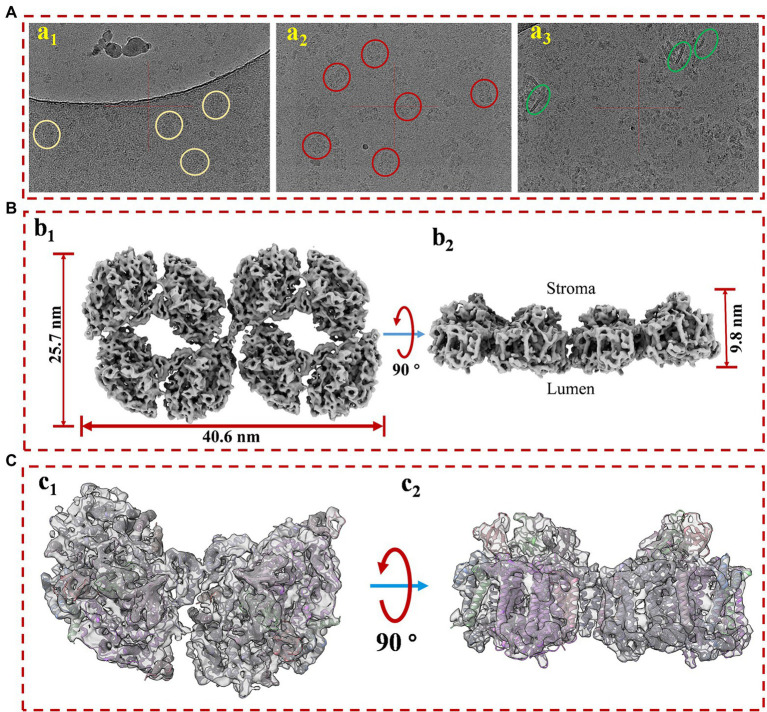
The cryo-EM sample prep optimization and structure determination of PSI supercomplex 2. **(A)** Optimization of cryo-EM sample. **(a**_**1**_**)** The protein particles were blotted immediately after sample loading; **(a**_**2**_**)** detergent fos-coline-8 was supplemented into the protein sample at a final concentration of 1.0 mM around 5 s before blotting; **(a**_**3**_**)** additional 3 s of waiting time were introduced between the loading and blotting of fos-choline-8 mixed sample. **(B)** Top **(b**_**1**_**)** and side **(b**_**2**_**)** views of the refined 3D density map of PSI supercomplex 2. **(C)** Model and map fitting for structure comparison of cyanobacterial PSI dimer and the supercomplex. Top **(c**_**1**_**)** and side **(c**_**2**_**)** views of model fitting of PSI dimer into the density map. The model used for fitting is a published PSI dimer structure (PDB code: 6K61) from *Anabaena* sp. PCC 7120.

In our experiments, the *in vitro* safety and stabilities of the PSI octamer are better than those of the hexamer; for this reason, a near-atomic resolution structure of stable PSI octamers was resolved by cryo-EM single-particle analysis in the present study. For maximal harvests and minimal destruction to the PSI octamer, the Native-PAGE-based purifying method was applied for sample purification. The whole process for concentrated pure samples preparation was completed in only 6–8 h, and the fresh molecules were then frozen immediately for cryo-EM single-particle analysis. Protein samples with a concentration of approximately 3 mg/ml were loaded onto the grids for frozen and quality evaluations. Initially, very few particles could be observed in the hole of the ice layer, while most of them clumped together around the carbon edge (Figure 2Aa_1_). To help particles move into the hole, an extra detergent, fos-choline 8, was added to the sample immediately just before the blotting. A significantly improved distribution of particles in the holes was obtained, with approximately 30 particles per micrograph (Figure 2Aa_2_). However, the presence of random orientations is a challenge when considering the surface charge of the complex. By optimizing the wait time between sample loading and blotting, we improved the distribution of the side view by approximately 10% (Figure 2Aa_3_). The preferential orientation issue is dependent on the biological specimen, and many options may proceed on a case-by-case basis (Tan et al., 2017; Han et al., 2020; Sorzano et al., 2021). If the particles are two linked PSI tetramers, then the preferential orientation is relatively inevitable as the hydrophilic area of the top view is as large as around 1,000 nm^2^, while it is less than 200 nm^2^ for the side view (Supplementary Figure 1). Additionally, top views have a much higher probability of being absorbed and face the air-water interface when a sample is blotted and frozen (Noble et al., 2018).

As revealed in Supplementary Figure 2, after 2D classification and screening, about 100 k good particles were randomly separated into 10 datasets and screened for 3D reconstruction and structure determination, respectively, in cryoSPARC. Reconstruction of this complex proceeded without applying symmetry (C1) from homogenous particle subsets. Non-uniform refinement from 12,121 particles of subset 5 generated a map at the overall resolution of 7.0 Å and the highest local resolution of 6.0 Å (Supplementary Figure 3). As shown in Figure 2B, the molecular sizes of PSI octamers are 40.6, 25.7, and 9.8 nm for length, width, and height, respectively. The overall shape of the map strongly suggested that this megacomplex is a PSI oligomer synclastic arranged by two tetramers linked side by side, which first proved that the complex is a PSI octamer structurally. To verify this conclusion, a PSI dimer model was fitted into a part of the map (Figures 2Cc_1_,c_2_). The secondary structures in both the transmembrane and out membrane region (lumen and stroma sides) can be fitted perfectly into the densities.

**Figure 3:**
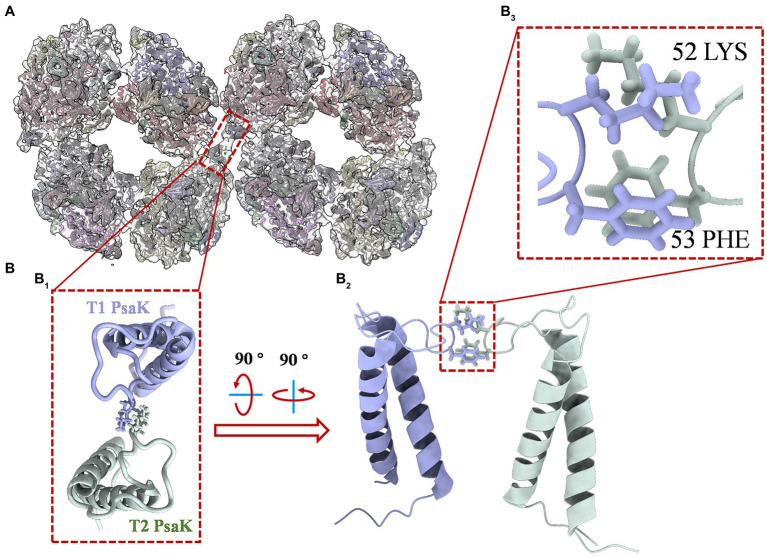
Structure details of PsaK-PsaK interaction in PSI octamer supercomplex. **(A)** Overall view of the interface location in the complex (top view from the stromal side). The original structure applied for fitting is the PSI tetramer model (PDB code: 6TCL) from *Anabaena* sp. PCC 7120. **(B)** Close-up view of structural details indicated in **(B**_**1**_**)**. The enlarged top view of two PsaK subunits from the assembled PSI tetramers (T1 and T2), the interacted amino acids were shown as stick form. **(B**_**2**_**)** Tilted side view of interacted PsaK subunits. **(B**_**3**_**)** The focus view of intact amino acids Lys52 and Phe53 in PsaK subunits.

Interestingly, a density of interface for linkage was objectively reconstructed in this map. As shown in Supplementary Figure 4, the density (red frame) is consistent with the threshold levels, and the firmness is stronger than the interfaces that form dimers (green frame) and tetramers (light blue frame). It is indicated that the newly identified PSI octamer is a physiologically relevant megacomplex rather than a random aggregation during sample preparation. This solid evidence was then obtained from the biochemical characterizations of PSI octamers from cells growing in changing physiological conditions. As shown in Supplementary Figure 5, by native-PAGE characterization of cells that incubated in different light conditions, the PSI octamer is a low-light responded supercomplex in *Anabaena* sp. PCC 7120. The result agrees with the conclusion from Wood’s work published in 2018 in which tighter packing of PSI complexes was detected in the dark (Wood et al., 2018). To find the possible interfaces for the assembly of two tetramers, separated PSI tetramer models were fitted rigorously into the map with the ChimeraX program. As shown in Figure 3, an interaction between PsaK subunits from two linked PSI tetramers was found in the fitted model (Figure 3A). As revealed in Figure 3B, two residues, Lys52 and Phe53, of PsaK from tetramer 1 (T1) interacted with the same residues from tetramer 2 (T2) with each other. This novel interface should be a major contribution to stabilizing PSI octamer. In summary, the map is qualified to be applied to perform rigid-body fitting with an atomic model when more structural details are required to be revealed. On the other hand, this map is reconstructed with datasets collected from the 200 kV microscope, and higher resolution is expected by using a zero-loss energy filter equipped detector. The resolution can also be theoretically significantly improved with a 300 kV microscope.

Higher-ordered PSI oligomers are physiologically relevant megacomplexes rather than random aggregations during sample preparation. For instance, in this study, the PSI octamer was strongly suggested as a low-light responded super complex in *Anabaena* sp. PCC 7120 (results shown in Supplementary Figure 5). A similar conclusion was presented by Wood’s observation through atomic force microscopy in which tighter packing of chloroplast PSI complexes is more abundant in the dark (Wood et al., 2018). And the super high-resolution structure of such a tight-packed chloroplast PSI oligomer was resolved very recently (Naschberger et al., 2021). A novel type of interface for connection between PSI monomers was structurally detected and genetically solidified. It has been demonstrated that the remodeling of photosynthetic machinery on the thylakoid membrane would happen in the low-light-adapted chloroplast (Wood et al., 2018). The dimeric PSII supercomplex has both a larger relative physical antenna size and higher relatively light-harvesting efficiency (Kirchhoff et al., 2007; Kim et al., 2020). And dimeric PSI-LHCI supercomplex was also found to be associated with higher cyclic electron transfer (Wood et al., 2018), which will help cells produce ATP more efficiently under stress. PSI-assembled heterogeneous or homogenous supercomplexes have frequently been detected biochemically in previous studies (Zhang et al., 2010; Järvi et al., 2011; Kouřil et al., 2018); here, we isolated two supercomplexes and set up biophysical characterizations of the molecules. Taking the evidence from low-temperature fluorescence and negative staining EM, we concluded that these two complexes are the PSI hexamer and PSI octamer, respectively. To our knowledge, this is the first study to isolate such higher-ordered PSI complexes in photosynthetic organisms, paving the way for a better understanding of the molecular architecture and physiological functions of each type of photosystem oligomers. Additionally, the cryo-EM structure of PSI octamer revealed a novel molecular interface (PsaK-PsaK) for PSI assembly in photosynthetic organisms.

## Data Availability Statement

The original contributions presented in the study are included in the article/Supplementary Material; further inquiries can be directed to the corresponding authors.

## Author Contributions

MC, PX, and YZ designed the research. YH, MC, DL, and QS performed the research and data curation under the supervision of LT and JH. MC wrote the manuscript with adding from YH, YP, and DL. YZ, LT, QS, and JH analyzed the data and edited the manuscript. JH and YZ supported the research with funding acquisition. All authors read and approved the manuscript.

## Funding

This work was supported by China Postdoctoral Science Foundation (grant no. 2020T130747), National Natural Science Foundation of China (31900429), Shenzhen Sanming Project of Medicine (SZSM201911003), Shenzhen Science Technology and Innovation Commission (SZSTI) Basic Research Program (JCYJ20190809165417340) and by 100 Top Talents Program of Sun Yat-sen University (grant no. 392009).

## Conflict of Interest

The authors declare that the research was conducted in the absence of any commercial or financial relationships that could be construed as a potential conflict of interest.

## Publisher’s Note

All claims expressed in this article are solely those of the authors and do not necessarily represent those of their affiliated organizations, or those of the publisher, the editors and the reviewers. Any product that may be evaluated in this article, or claim that may be made by its manufacturer, is not guaranteed or endorsed by the publisher.
